# An unusual necrotic myositis by *Clostridium perfringens* in a German Shepherd dog: A clinical report, bacteriological and molecular identification

**Published:** 2015-12-15

**Authors:** Hamideh Salari Sedigh, Masoud Rajabioun, Jamshid Razmyar, Hossein Kazemi Mehrjerdi

**Affiliations:** *Department of the Clinical Sciences, Faculty of Veterinary Medicine, Ferdowsi University of Mashhad, Mashhad, Iran.*

**Keywords:** *Clostridium perfringens*, Dog, Necrotic myositis

## Abstract

Clostridial myositis, considered to be rare in pet animals, is an acutely fatal toxaemic condition. Some species of clostridia are responsible for necrotic myositis. A 2-year-old male German shepherd dog was admitted with non-weight bearing lameness and massive swelling of the left hind limb. *Clostridium perfringens* type A with alpha toxin was diagnosed as a pathogenic agent. Based on the history, the bacteria were introduced inside the tissue via contaminated needle following intramuscular injection. Urgent medical therapy followed by surgical intervention was performed. The dog was discharged completely healthy after hospitalization for four weeks. The objective of this report was to describe necrotic myositis in a dog with an emphasis on clinical signs and treatment as well as bacteriological and molecular identification of the micro-organism. Because of the fatal entity of the disease, prompt diagnosis as well as proper and urgent treatment is very important for successful therapy.

## Introduction

Clostridia are spore forming, Gram-positive, anaerobic, encapsulated bacilli of the genus clostridium discovered by Lucey *et al*. Welch in 1981.^1^ These bacteria can be the cause of significant diseases and sometimes death both in humans and animals.^2^ More than 150 species of clostridia have been identified but the most commonly isolated is *Clostridium perfringens* type A (95.0%) either alone or in combination with other pathogenic clostridia, *Clostridium novyi* (8.0%), *C. septicum* (4.0%), *C. histolyticum*, *C. fallax*, and *C. sordelli* (1.0% or less of the infections). Clostridia are saprophytic found in soil, dust, water and the intestine of humans and animals. Six species of clostridia have been reported to cause gas gangerene in humans: *C. perferinges*, *C. novyi*, *C. septicum*, *C. histolyticum*, *C. bifermentans* and *C. fallax*. More than 80.0% of cases of gas gangrene are caused by *C. perferingens*.^3^

*Clostridium perfringens* is classified into five toxin types (A-E) according to the production of four major toxins, namely alpha (cpa), beta (cpb), epsilon (etx) and iota (itx). *Clostridium perfringens* causes numerous gastro-intestinal infections in most mammalian species. This micro-organism can also cause diseases of skin, subcutaneous and muscular tissues (gas gangrene or malignant edema). Most of diseases produced by *C. perfringens* are mediated by one or more of its powerful toxins.^4^^-^^5^

There are several reports of myonecrosis caused by clostridia in dogs, but in most of them have been presented only clinical and postmortem data, without potent etiological identification.^6^

This report described a treated case of necrotic myositis by *C. perfringens* in a dog caused by contaminated needle used for administering drug as well as bacteriological and molecular identification of the pathologic agent.

## Case Description

A 2-year old male German Shepherd dog was admitted into the Veterinary Teaching Hospital, School of Veterinary Medicine, Ferdowsi University of Mashhad for non-weight bearing lameness and massive swelling of the left hind limb in the thigh region. According to the history taken from the owner, the swelling had begun from the previous two days when the dog was given an intramuscular anesthetic injection for restraint during purchase.

**Fig. 1:**
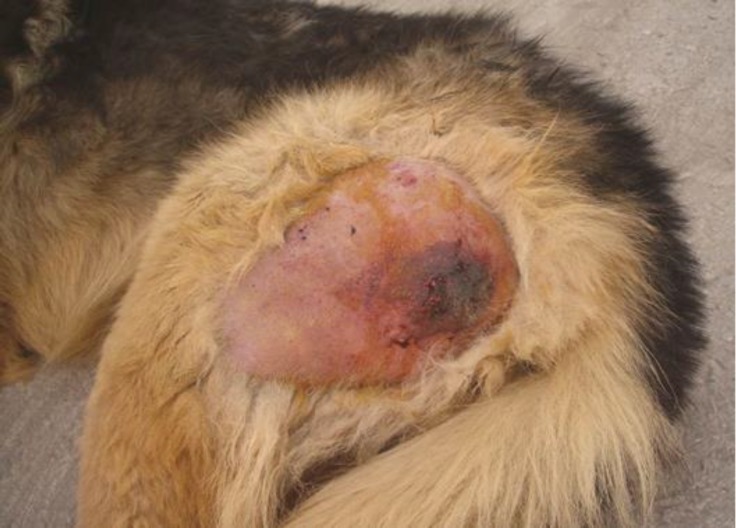
Lateral view of the left hind limb after removal of hair on initial examination. Note the soft tissue swelling and discoloration of the skin into dark-red color at the caudal aspect of the thigh (The site of the intramuscular injection

Initial examination revealed fever (41.9 ˚C), tachypnea (respiratory rate = 60 bpm), normal heart rate (96 bpm) and diffuse non-pitting edema of the left pelvic limb around the left thigh. The left limb was massively swollen and tense, displaying severe cracking and soreness upon palpation, which indicated the presence of gas and edema. After shaving the affected area, a dark-red-rounded discoloured skin was seen on the caudal aspect of the left thigh that was well demarcated from the surrounding healthy skin (Fig. 1). Furthermore, the animal showed weakness, dyspnea and fever (41.2 ˚C). Manipulation of the limb was extremely painful, but no other injuries were apparent. Blood sample was taken and submitted for a complete blood count, creatine phosphokinase and liver enzymes level. Samples of tissue secretions were taken by sterile syringe for bacterial culture and antibiogram test.

**Microbiological examination.** Bacterial culture was carried out and incubated overnight in anaerobic conditions at 37 ˚C on blood agar media containing 5.0% sheep blood. Further passages from single colonies were sub-cultured on blood agar plates (Merck, Darmstadt, Germany) incubated overnight in anaerobic conditions using gas pack A jar (Merck, Darmstadt, Germany) at 37 ˚C. 

A single colony of the strain was suspended in 100 µL distilled water, boiled for 10 min and then centrifuged at 10000 g for 10 minutes. The supernatants were collected carefully and used as template DNA for polymerase chain reaction (PCR). Six pairs of primers were used to determine the presence of cpa, cpb, iA, etx, cpe and cpb2 genes using a multiplex PCR technique for the isolate. The primers were provided by DENAzist Asia Co. (Mashhad, Iran) and other materials used in PCR reaction were provided by Ampliqon (Odense, Denmark). The procedure was carried out according to previously published work.^6^

Bacterial culture revealed pure culture of *C. perfringens* and molecular typing of the isolates Type A (cpb2+) and positive for cpa toxin gene. Based on antibiogram test, the isolate was resistant to cefazolin, florfenicol and ceftriaxone but susceptible to penicillin, sulfadimidine, sulfadiazine and neomycin. 

Minimum inhibitory concentrations were determined by microdilusion method based on Clinical and Laboratory Standards Institute (CLSI) protocols on the *Brucella* broth (Hi-media, Mumbai, India) plates containing doubling dilutions of the antibiotics, from 0.25 to 0.256 mg L^-1^, and supplemented with 5.0% sheep blood. Prior to anti-microbial susceptibility testing, isolates were sub-cultured on a tioglicolate broth (Himedia, Mumbai, India). After incubation in anaerobic atmosphere for 18 hours at 37 ˚C, the cultures were suspended in a 0.85% NaCl to an optical density equivalent to that of a 0.5 McFarland standard. The isolate was inoculated on 96 wells plate. Reactions were tested twice and every test plates included positive and negative controls. 

**Fig. 2:**
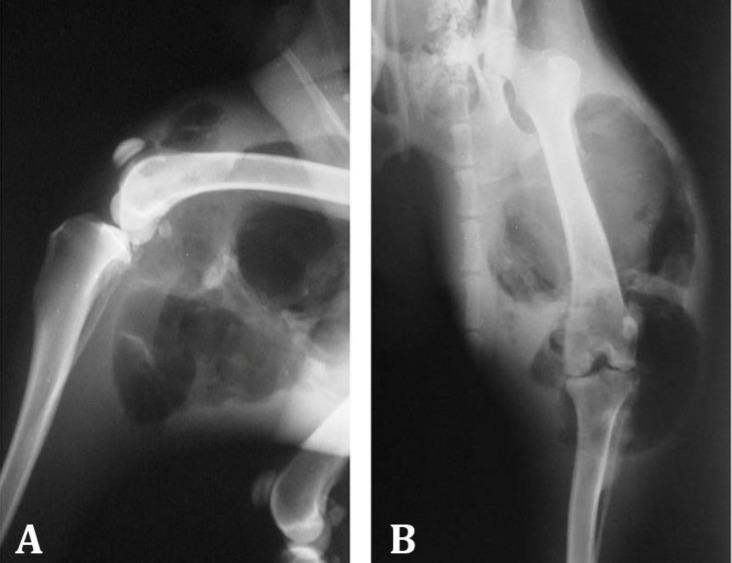
Lateral (A) and cranio-caudal (B) radiographs of the left hind limb. Note the severe soft tissue swelling and severe gas accumulation in soft tissue around the femur and stifle joint

**Treatment and outcome. **The therapy was performed initially based on aggressive fluid therapy, subcutaneous enrofloxacin (10 mg kg^-1^; Razak Laboratories Co., Tehran, Iran) and intravenous metronidazole (15 mg kg^-1^; Samen Pharmaceutical Co., Mashhad, Iran). After the emergency treatment the patient was referred to radiology ward and lateral and craniocaudal radiographs of the affected limb were taken. The radiographs revealed severe soft tissue swelling with presence of massive gas in soft tissues around the left thigh and stifle joint indicative of emphysema with-out any sign of bone and joint involvement. Gas trapping was also seen in caudal aspect of the left femur and stifle joint (Fig. 2). Hematology revealed severe leukocytosis (31300 cells per µL) and neutrophilia (adult: 25040 cells per µL, band: 2817 cells per µL) resulting from sepsis and biochemistry panel showed elevation of liver enzymes (alanine aminotransferase: 137 U L^-1^, aspartate amino-transferase: 349 U L^-1^, alkaline phosphatase: 420 U L^-1^) and also creatine phosphokinase (6007 IU L^-1^) indicating muscle injury.

The dog was hospitalized and treatment was continued by the penicillin (50000 IU kg^-1^) based on the result of fine-needle aspiration cytology that indicatedthe presence of spores. Two days after initial presentation, the animal remained non-weight bearing and discoloration area was enlarged indicating the progress of the lesion.

The dog was referred to surgery ward and after premedication with intramuscular acepromazine (0.03 mg kg^-1^; Alfasan, Woerden, The Netherlands), anesthesia was induced with combination of intravenous diazepam (0.2 mg kg^-1^; Caspian Tamin Pharmaceutical Co., Rasht, Iran) and ketamine (6 mg kg^-1^; Alfasan, Woerden, The Netherlands). Following intubation, anesthesia was maintained using 1.5 to 2% isoflurane (Nicholas Piramal Ltd., Mumbai, India) delivered in 100% oxygen. A lengthwise incision was made in the relevant area of the affected hindlimb to allow debridement and resection of the necrotic tissue extending through the muscles and pus. A large quantity of pink, foul-smelling, purulent fluid was removed. The tissues were lavaged with 500 mL of 5% oxygenated water for five days after operation, and then before wound closure, a penrose drain was inserted subcutaneously. Postoperative pain was controlled by injectable meloxicam (0.3 mg kg^-1^, Razak Laboratories Co., Tehran, Iran) intramuscularly for three days.

Postoperative follow-up revealed satisfactory improve-ment, food and water were taken on the day after surgery, urine and faeces were passed and the temperature fell to normal range within 48 hr. Based on culture and antibiogram test, 4.5 g piperacillin/tazobactam (Daana Pharma Co., Tehran, Iran) were injected intravenously as continuous rate infusion in Nacl 0.9% dextrose 5.0% solution every 12 hr for three days. After that ampicillin (25 mg kg^-1^, Jaber-Ebne-Hayyan Pharmaceutical Co., Tehran, Iran) was given orally every 8 hr for four weeks and then 5.0% sodium hypochlorite dip used every other day to reduce anaerobic clostridia count. The dog was hospitalized for four weeks and discharged completely healthy.

## Discussion

Clostridial myositis is often recognized as an acute, rapidly progressive, invasive edema, massive death of tissue and a variable degree of gas production, reported in cattle,^2^^-^^7^ horses^,^^9^^-^^13^ sheep and goats,^14^ pigs,^15^ black bear,^16^ rhesus macaque,^17^ and dog.^18^^-^^23^ Cases have been reported in the literature in association with orthopaedic injuries, intra-muscular injections or with no apparent injury.^18^^-^^22^^,^^23^^-^^26^ The initial source of the infection was not identified in some reported cases.^18^^,^^21^^-^^23^ In this report, infection was trans-mitted most likely through the infected injection needle.

The majority of clinical cases of clostridial myositis have been associated with *C. perfringens*, but infections with *C. septicum* and *C. chauvoei* have also been reported.^22^^,^^26^

Typically, clostridial infections are characterized by muscle necrosis with gas and toxin production, and associated with foul smelling exudates. The affected limb is swollen and painful. The patient is usually pyrexic with varying signs of toxaemia including congested mucous membranes, weak pulse rate, and elevated heart and respiratory rates.

In the present reported case, the *C. perfringens* isolates were Type A and positive for the cpa toxin gene, genotyped using multiplex PCR.^27^ Type A of* C. perfringens* is a normal resident of the environment and illness attributed to *C. perfringens* has been described.^28^ Alpha toxin has been shown to affect myocardial contractility, causing hypotension and bradycardia, resulting in shock, a common and often fatal feature of gas gangrene. The toxin also increases vascular permeability, as can be demonstrated by intradermal injection.^29^ Several reports described canine clostridialmyonecrosis without molecular characterization of the bacteria.^5^^,^^18^^,^^19^^,^^21^^,^^23^^,^^24^ Ribeiro *et al*. identified *C. Septicum* as a cause of myonecrosis in dog by molecular examination.^22^

In humans, clostridial myonecrosis or gas gangrene is a severe infection infrequently encountered by most physicians. Early clinical recognition and treatment considerably alter the outcome. In untreated patients, the disease is usually fatal.^3^

Clostridial myonecrosis has two major presentations in humans: spontaneous and nonspontaneous. Historically, the vast majority of cases of clostridial myonecrosis, also known as gas gangrene, were nonspontaneous and seen in the setting of penetrating trauma with contamination of the deep muscle compartments from spores present in the soil. Traumatic gas gangrene is caused by *C. perfringens*, an anaerobic spore-forming gram-positive *Bacillus*. Patients are presented with rapid onset of severe pain, skin dis-coloration, fulminant tissue destruction, and gas formation in the soft tissues after penetrating trauma. If left untreated, sepsis, multi-organ failure, and death rapidly ensue.^30^

Currently employed treatments include surgery, antibiotics, and hyperbaric oxygen. The use of radio-therapy for the treatment of patients with gas gangrene has also been documented.^31^

Based on the Practical guideline for humans updated by infectious disease society of America, urgent surgical exploration of the suspected gas gangrene site and surgical debridement of involved tissue should be performed and in the absence of a definitive etiologic diagnosis, broad-spectrum treatment with vancomycin plus either piperacillin/tazobactam, ampicillin/sulbactam, or a carbapenem antimicrobial is recommended. Hyperbaric oxygen therapy is not recommended because it has not been approved of benefit to the patient and may delay resuscitation and surgical debridement.^32^

In human literature, necrotizing soft-tissue infections can be classified according to depth of tissue involvement, severity of infection, or microbiology. Each system has its advantages. The Food and Drug Administration classifies infections of skin and soft tissues as either complicated or uncomplicated. An uncomplicated infection responds to a simple course of antibiotics or incision and drainage.

Complicated infections involve deeper tissues and generally require surgical intervetions.^33^^-^^34^ Because of life**-** threatening nature of this infectious, rapid diagnosis and treatment must be considered by clinician for saving the dog.
